# Dual Imaging Gold Nanoplatforms for Targeted Radiotheranostics

**DOI:** 10.3390/ma13030513

**Published:** 2020-01-22

**Authors:** Francisco Silva, António Paulo, Agnès Pallier, Sandra Même, Éva Tóth, Lurdes Gano, Fernanda Marques, Carlos F.G.C. Geraldes, M. Margarida C.A. Castro, Ana M. Cardoso, Amália S. Jurado, Pilar López-Larrubia, Sara Lacerda, Maria Paula Cabral Campello

**Affiliations:** 1 Centro de Ciências e Tecnologias Nucleares, Instituto Superior Técnico, Universidade de Lisboa, Campus Tecnológico e Nuclear, Estrada Nacional 10, Km 139.7, 2695-066 Bobadela LRS, Portugal; fsilva@ctn.tecnico.ulisboa (F.S.); apaulo@ctn.tecnico.ulisboa.pt (A.P.); lgano@ctn.tecnico.ulisboa.pt (L.G.); fmarujo@ctn.tecnico.ulisboa.pt (F.M.); 2 Departamento de Engenharia e Ciências Nucleares (DECN), Instituto Superior Técnico, Universidade de Lisboa, Estrada Nacional 10, 2695-066 Bobadela LRS, Portugal; 3 Centre de Biophysique Moléculaire, CNRS, UPR 4301, Université d’Orléans, Rue Charles Sadron, 45071 Orléans CEDEX 2, France; agnes.pallier@cnrs.fr (A.P.); sandra.meme@cnrs.fr (S.M.);; 4 Department of Life Sciences, Faculty of Science and TechnologyUniversity of Coimbra, Calçada Martim de Freitas, 3000-393 Coimbra, Portugalasjurado@bioq.uc.pt (A.S.J.); 5 Coimbra Chemistry Center, University of Coimbra, 3004-535 Coimbra, Portugal; 6 CIBIT/ICNAS Instituto de Ciências Nucleares Aplicadas à Saúde. Pólo das Ciências da Saúde, Azinhaga de Santa Comba, 3000-548 Coimbra, Portugal; 7 CNC-Center for Neuroscience and Cell Biology, University of Coimbra, 3004-517 Coimbra, Portugal; amscardoso@ci.uc.pt; 8 Institute for Interdisciplinary Research of the University of Coimbra, 3030-789 Coimbra, Portugal; 9 Instituto de Investigaciones Biomédicas “Alberto Sols” CSIC/UAM, c/ Arturo Duperier 4, 28029 Madrid, Spain; plopez@iib.uam.es

**Keywords:** gold nanoparticles, multimodality, MRI, SPECT, bombesin, PC3 tumor, radiosensitization

## Abstract

Gold nanoparticles (AuNPs) are interesting for the design of new cancer theranostic tools, mainly due to their biocompatibility, easy molecular vectorization, and good biological half-life. Herein, we report a gold nanoparticle platform as a bimodal imaging probe, capable of coordinating Gd^3+^ for Magnetic Resonance Imaging (MRI) and ^67^Ga^3+^ for Single Photon Emission Computed Tomography (SPECT) imaging. Our AuNPs carry a bombesin analogue with affinity towards the gastrin releasing peptide receptor (GRPr), overexpressed in a variety of human cancer cells, namely PC3 prostate cancer cells. The potential of these multimodal imaging nanoconstructs was thoroughly investigated by the assessment of their magnetic properties, in vitro cellular uptake, biodistribution, and radiosensitisation assays. The relaxometric properties predict a potential T_1_- and T_2_- MRI application. The promising in vitro cellular uptake of ^67^Ga/Gd-based bombesin containing particles was confirmed through biodistribution studies in tumor bearing mice, indicating their integrity and ability to target the GRPr. Radiosensitization studies revealed the therapeutic potential of the nanoparticles. Moreover, the DOTA chelating unit moiety versatility gives a high theranostic potential through the coordination of other therapeutically interesting radiometals. Altogether, our nanoparticles are interesting nanomaterial for theranostic application and as bimodal T_1_- and T_2_- MRI / SPECT imaging probes.

## 1. Introduction

The primary source of information for clinical diagnostic applications comes from medical imaging. The available molecular imaging modalities provide different information about the patient with different spatial and temporal resolution and sensitivity. For that reason, it is currently accepted that a single imaging modality cannot provide sufficient and exact information of the human body. Besides that, nowadays there is an increasing interest in merging diagnostic and therapeutic functions in the same tool to perform the so-called “theranostics” [1,2,3,4]. Nanotechnology is now a central field in biomedical research, namely to design nanosized theranostic agents. Due to their unique optical properties, non-toxic nature, relatively simple preparation, and functionalization, gold nanoparticles (AuNPs) are excellent candidates for this type of application. AuNPs are versatile nanoplatforms that can be easily functionalized with several imaging agents, plus biological targeting moieties and drugs, combining therapeutic and diagnostic functions within a single nanoparticle [4,5,6,7,8]. While this concept is auspicious, AuNPs have to overcome multiple hurdles to reach their full potential in clinical applications. The limitations in medical practice are closely associated with the pharmacokinetics and tissue distribution of AuNPs that are strongly dependent on their physical and chemical properties, and surface composition. Thus far, the real “magic gold nanoparticle” still remains an unmet goal despite the recent and encouraging results that have been reported for target-specific AuNPs applied to cancer theranostics. Indeed, this progress is only at the proof-of-concept stage and its application to clinical trials is still a few years away [9,10,11].

Magnetic Resonance Imaging (MRI) is one of the most widely used imaging modalities in clinics. It often makes use of the paramagnetic properties of Gd^3+^ based Contrast Agents (GBCAs) to enhance image contrast, due to their reduction of water proton relaxation times of selected tissues. MRI provides anatomical images inside the human body with high spatial resolution, although with relatively low sensitivity [12,13,14,15,16]. The Solomon–Bloembergen–Morgan (SBM) theory predicts that very high relaxivities, of the order of magnitude 100 mM^−1^·s^−1^at magnetic fields relevant for clinical imaging, are attainable by Gd^3+^ chelates displaying simultaneous optimization of the main parameters that govern relaxivity: Rotational correlation times (τ_R_), water exchange rate constant (k_ex_), and electron spin relaxation parameters (τ_v_ and Δ^2^) [12,17,18,19]. However, despite many attempts, this goal is still elusive. In fact, the clinically approved low molecular weight GBCAs display relaxivities of the order of magnitude 3–5 mM^−1^·s^−1^at magnetic fields relevant (currently) for clinical MRI (20–120 MHz). However, nuclear imaging techniques, like Single Photon Emission Computed Tomography (SPECT) and Positron Emission Tomography (PET), provide physiological images that contain information about metabolic parameters with high sensitivity, but have low spatial resolution [20,21,22]. Several in vivo MRI studies established the merits of functionalized AuNPs as CAs for MRI, bimodal MRI/X-ray imaging, and as theranostics platforms. More recently, in the late twentieth century, due to the high cross-section of the photoelectric effect in gold, gold nanoparticles have been evaluated in vitro and in vivo as enhancers of the radiation used in cancer therapy. Studies have shown that the synergistic effect of irradiation and gold nanoparticles can lead to a significant tumor reduction. Moreover, the benefit of this combinatory therapy is also strongly dependent on the size, coating, and number of internalized nanoparticles in cancer cells and on the radiation energy [23,24,25,26,27,28,29,30,31,32,33,34]. A few Gd^3+^-based molecules and particles have also been reported, combining MRI guided imaging with radiosensitization. Indeed, Gd^3+^ is well known for its paramagnetic properties, but its high mass absorption coefficient and high atomic number also makes it interesting for such applications [28,35]. Up to the present time, there are no Gd^3+^-bearing AuNPs that have been evaluated in clinical trials for MRI, and PET/MRI bimodal imaging studies are in general scarce [36,37,38,39]. While some studies have been done regarding the application of AuNPs for theranostics, their therapeutic function is mostly based on the photothermal properties of the AuNPs themselves or on the delivery of a payload acting as a cytotoxic drug [40,41,42,43,44,45,46]. There are some examples of AuNPs that have been evaluated in the delivery of medically relevant radionuclides, aiming to obtain “radiotheranostic” agents suitable to provide clear and sensitive tumor detection and selective therapeutic effects [47,48]. This included nanoconstructs decorated with bombesin (BBN) analogues, a bioactive peptide with high affinity towards the Gastrin Releasing Peptide receptor (GRPr) overexpressed in a variety of human cancers, and demonstrated that the AuNPs displayed specificity towards GRPr overexpressing tumors [49,50,51].

Within our on-going studies on GRPr-targeted nanotools, we have evaluated some radiolabeled AuNPs stabilized with an acyclic DTPA derivative (DTTPA = (2-[bis[2-[carboxymethyl-[2-oxo-2-(2-sulfanylethylamino)ethyl]amino]ethyl]amino]acetic acid) or with a macrocyclic DOTA derivative (TDOTA = trimethyl 2,2′,2″-(10-2(3-(tritylthio)propamido)ethyl)-1,4,7,10-tetraazacyclododecane-1,4,7-trityl)triacetate), and functionalized with BBN analogue peptides for specific tumor targeting. These studies proved that the DOTA-containing AuNPs functionalized with a BBN thioctic derivative are the most promising ones, allowing for a fast complexation of trivalent radiometals (*e.g*. ^67^Ga^3+^) and showing high in vitro/in vivo stability, along with a favorable cellular internalization in GRPr-positive cells and significant tumor uptake [52,53]. Herein, we describe our efforts to assess the capacity of these BBN-containing AuNPs to anchor, at the same time, trivalent metal ions adequate for MRI (e.g., Gd^3+^) and for radioimaging (e.g., ^67^Ga^3+^), taking advantage of the favorable coordination properties of the DOTA-based chelator towards Gd^3+^ and Ga^3+^, yielding a bimodal MRI/nuclear imaging reporter. This study encompassed the determination of the total amount of Gd^3+^ that can be loaded into the AuNPs, while still allowing for the possibility of having free DOTA unities available for further coordination of radiometals. The physico-chemical characterization of the resulting Gd-AuNPs, including their relaxometric properties, is also described. Cell uptake studies in PC3 human prostate cancer cells were performed to confirm that the inclusion of the Gd^3+^ ions did not compromise the GRPr-mediated cellular uptake. The biodistribution was assessed in tumor-bearing mice and healthy animal models, through ^67^Ga^3+^ measurements in main organs. As proof of the principle, the potential for radiosensitization of these gadolinium-loaded AuNPs was also evaluated. These studies will contribute to a better understanding of the biological performance of these AuNPs as potential bimodal MRI/nuclear imaging probes, and of their relevance as novel nanomaterials for cancer theranostics.

## 2. Materials and Methods 

### 2.1. General Procedures

All chemicals and solvents were of reagent grade and were used without further purification, unless stated otherwise, and were commercially acquired from Aldrich Chemical Co. (Saint Louis, MO, USA). Solvents for high-performance liquid chromatography (HPLC) were HPLC-grade. For the preparation of aqueous solutions and for rinsing of gold nanoparticles, Milli-Q (DI) water (ρ < 18 MΩ) was used. The amino acids used in this work were acquired from Novabiochem. Anhydrous GdCl_3_ was obtained from Alfa Aesar. AuNP-TDOTA and thioctic acid terminated bombesin peptide (TA-BBN) were synthesized according to previously published methods [53]. ^67^GaCl_3_ was prepared from ^67^Ga-citrate (acquired from Mallinckrodt) following a protocol previously described [54]. The PC3 cell line was obtained from ATCC, Manassas, VA, USA.

Ethics statement. Animal studies were carried out under the supervision of authorized researchers in laboratory animal facilities licensed by the National Authority in compliance with the principles of laboratory animal science on animal care, protection and welfare and are properly accredited by the respective National Authorities according to the national (DL 113/2013) and EU (Directive 63/2010/EU) legislation for laboratory animal experimentation. The research project was also approved by the local ethical committee (ORBEA of IST-ID).

### 2.2. Synthesis of AuNP-TDOTA

AuNPs were synthesized according to our previously described method [53]. Briefly, TDOTA·2TFA (68 mg, 0.10 mmol) was dissolved in 10 mL of a MeOH/H_2_O solution (7:3), and added to 4.5 mL of a solution of HAuCl_4_·3H_2_O (1 mM) in MeOH. The mixture was stirred at room temperature for 5 min and then 0.3 mL of a solution of NaBH_4_ (0.295 M) was added while vigorously stirring. There was an immediate formation of a dark precipitate. The reaction was stirred for an additional hour at room temperature. The solution was centrifuged at 1000 rpm for 20 min and the pellet obtained was washed two times with MeOH and two times with H_2_O. The nanoparticles were then lyophilized and stored until further use.

### 2.3. Synthesis of AuNP-Gd

To 5 solutions of AuNP-TDOTA (600 µL, 5 mg/mL) different amounts of GdCl_3_ were added: 3.7 mg (14.0 × 10^−3^ mmol), 0.37 mg (1.4 × 10^−3^ mmol), 0.185 mg (7.0 × 10^−4^ mmol), 0.074 mg (2.8 × 10^−4^ mmol), and 0.037 mg (1.4 × 10^−4^ mmol) in 1 mL of H_2_O. The mixtures were stirred overnight at room temperature and then ultra-centrifuged using an Amicon 10k, washed with H_2_O, lyophilized, stored at −20 °C, and restored in H_2_O prior to use.

### 2.4. Synthesis of AuNP-Gd-BBN

200 µL of MeOH was added to a solution of AuNP-Gd (200 µL, 5 mg/mL, prepared using 1:0.25 Au/Gd ratio), followed by the addition of a TA-BBN solution (2 mg (0.0018 mmol)) in 400 µL of MeOH. The mixture was stirred overnight at room temperature and then ultra-centrifuged using an Amicon 10k and washed with MeOH and finally with H_2_O. The nanoparticles were lyophilized and stored at −20 °C.

### 2.5. Determination of the Amount of Conjugated TA-BBN

Determination of TA-BBN payload was performed using a HPLC procedure previously reported [53]. Solutions of TA-BBN were prepared, and split into equal parts, one part of the peptide solution was used for the reaction with AuNP-Gd and the other part used to prepare a control solution. The control solution was diluted in order to have the same volume as the reaction mixture. The control solution and the supernatant of the reaction mixtures were analyzed by HPLC using identical equipment parameters. The difference in the area under the curve was used to determine the amount of conjugated peptide (Area (control, µV/s) = 2874925.66; Area (supernatant, µV/s) = 1724932.98). 0.8 mg of TA-BBN was conjugated to 1 mg of AuNP-Gd.

HPLC analyses were performed in a Perkin-Elmer LC200 pump with a UV-Visible Shimadzu LC290 and a Berthold LB-507A γ-detector, using a Macherey-Nagel EC 250/4 Nucleaosil 100-10 C18 (or 100-5 C18) with a flow rate of 1.0 mL/min (or 0.5 mL/min for column 100-5 C18).

### 2.6. Determination of Gadolinium and Gold Content by Inductively Coupled Plasma Optical Emission Spectroscopy (ICP-OES)

Spectrometric (ICP-OES) measurements were performed in a Jobin Yvon ULTIMA2 Spectrometer (Longjumeau, France). Both Gd and Au standard solutions were prepared in 5% HNO_3_ matrix. The NP samples were digested in conc. HNO_3_ for 48 h at room temperature followed by 18 h at 65 °C. The resulting solutions were then diluted 1:12, to reach a 5% HNO_3_ concentration. The quantitative determinations were performed using the most accurate band: 310.050 nm for Gd and 242.795 nm for Au. All the measurements were done in triplicate.

### 2.7. Dynamic Light Scattering (DLS) and Zeta Potential Determination

DLS measurements were performed with a Malvern Zetasizer Nano ZS (Malvern Instruments Ltd., Worcestershire, UK) equipped with a 633 nm He-Ne laser and operating at an angle of 173°. The software used to collect and analyze the data was the Dispersion Technology Software (DTS) version 5.10 from Malvern. 600 µL of each sample was measured in low volume semi-micro disposable sizing cuvettes (Fisher Scientific, USA) with a path length of 10 mm. Triplicate measurements were made at a position of 4.65 mm from the cuvette wall with an automatic attenuator. For each sample, 15 runs of 10 s were performed. The size distribution, the Z-average diameter and the polydispersity index (PDI) were obtained from the autocorrelation function using the “general purpose mode” for all nanoparticle samples. The default filter factor of 50% and the default lower threshold of 0.05 and upper threshold of 0.01 were used. Zeta potential measurements were performed in triplicates using water as a dispersant and the Huckel model. For each sample, 20 runs were performed in auto analysis mode.

### 2.8. Transmission Electron Microscopy

Transmission electron microscope images were obtained on a JEOL 1400 transmission electron microscope (TEM), JEOL LTD., Tokyo, Japan. TEM samples were prepared by placing 5 µL of gold nanoparticle solution on the 300-mesh carbon coated copper grid. Excess solution was removed carefully and the grid was allowed to dry for an additional five minutes. The average size and size distribution of the nanoparticles were determined by processing the TEM image Adobe Photoshop with Fovea plug-ins.

### 2.9. Radiolabeling with ^67^Ga^3+^

20 µL of NP solutions (5 mg/mL in DI water) was mixed with 350 µL of 0.4 M ammonium acetate (pH ≈ 7). To this mixture, 250 µL of ^67^GaCl_3_ (0.1 M HCl) was added, followed by heating at 70 °C for 30 min. After cooling to room temperature, the reaction mixture was then filtered through a Millipore Amicon Ultra 0.5 mL 10k. The collected gold nanoconstructs were diluted in 150 µL of H_2_O and their radiochemical purity assessed by ITLC-SG, using 6M HCl/MeOH (5:95) as an eluent. All nanoparticles were obtained with a final radiochemical purity >95%.

### 2.10. NMRD Profile

The *r*_1_ nuclear magnetic relaxation dispersion (NMRD) profiles of the Gd^3+^ containing particles were recorded on a Stelar SMARtracer FFC fast-field-cycling relaxometer covering magnetic fields from 2.35 × 10^−4^ T to 0.25 T, which corresponds to a proton Larmor frequency range of 0.01–10 MHz. The relaxivity at higher fields was measured using a Bruker WP80 adapted to variable field measurements and controlled by the SMARtracer PC-NMR console. The temperature was controlled by a VTC90 temperature control unit, fixed by a gas flow, and calibrated with a Pt resistance temperature probe. The concentration of the samples was determined by ICP-OES.

### 2.11. MRI Phantoms

MR images of tubes containing PBS, AuNP-Gd and AuNP-Gd-BBN were acquired on a BioSpec 9.4 T horizontal magnet (94/21 USR, Bruker BioSpin, Wissembourg, France) equipped with B-GA06S gradient system (950 mT/m maximal strength and 35 mm inner diameter) and Paravision 5.0 software (Bruker BioSpin, Wissembourg). Images were acquired using rapid spin echo sequence (RARE sequence, factor 8) with TE = 10 ms; 30 ms; 50 ms; 70 ms; and 90 ms and TR = 2.5 s; 1.5 s; 1.0 s; 0.8 s; 0.5 s; and 0.2 s. The resolution was 156 × 156 μm^2^ with a matrix of 256 ×256. Slice thickness was 1.0 mm. T_1_ and T_2_ maps were generated with Paravision software, and T1 and T2 values were calculated from these maps. The Gd^3+^ concentrations were assessed by ICP-OES.

### 2.12. Cytotoxicity Studies

PC3 cells were plated in 96-well plates at a density of 3 × 10^5^ cells per well. Twenty-four hours after plating, the cells were incubated with 1, 2.5, 5, 10, 20, 40, 80, and 160 µg/mL solutions of AuNPs (solutions prepared in RPMI complete medium). Cell viability was measured after 4 or 24 h using the modified Alamar Blue colorimetric assay [55]. Cell viability was presented relative to the percentage of untreated cells (control cells) considered as 100%. Data represent the mean ± SD of four independent experiments (Figure S1).

### 2.13. Cellular Internalization Studies

Internalization assays of the radiolabeled AuNPs were performed in PC3 cells seeded at a density of 2 × 10^5^ cells in 0.5 mL of culture medium per well in 24 well-plates and were allowed to attach overnight. The cells were incubated at 37 °C for a period of 15 min to 3 h with about 400,000 cpm of the ^67^Ga-AuNPs in 0.5 mL of culture medium. Incubation was terminated by washing the cells with ice-cold medium. Cell surface-bound radioactivity was removed by two steps of acid wash (50 mM glycine, HCl/100 mM NaCl, pH 2.8) at room temperature for 4 min. The pH was neutralized with cold PBS, and subsequently the cells were lysed by 10 min incubation with 1 M NaOH at 37 °C to determine the internalized compound. The radioactivity associated to each fraction in the cells was measured in a gamma counter (LB2111, Berthold, Germany) and were expressed as the percentage of the total activity added to the cells and presented as an average plus the standard deviation of three independent experiments.

After radioactive decay of the ^67^Ga (10 half-lifes), samples of the cells incubated for 3 h with the different ^67^Ga-labelled AuNPs were digested and their Gd amount was determined by ICP-OES (see the detailed ICP protocol above).

### 2.14. Irradiation Setup

PC3 cells were seeded in 96-well plates at a density of 3 × 10^4^ cells per well. The next day, the cells were incubated with 0.6, 4.6, 18.7, 37.5, and 75 µg/mL solutions of AuNPs in RPMI complete medium for 3 h. Cells were then irradiated with gamma-rays (1530 keV) using a ^60^Co irradiator (AECL Medical Products) at a cumulative dose of 2 Gy with a field size of 20 × 20 cm. After irradiation, the medium was removed and replaced by fresh medium. Cell viability was measured by the colorimetric MTT (3-(4,5-dimethylthiazol-2-yl)-2,5-diphenyltetrazolium bromide) assay after 72 h incubation in fresh medium. At the end of incubation, the medium was discarded and the cells were incubated with 200 µL of an MTT solution in PBS (0.5 mg/ml). After 3 h at 37 °C, the medium was removed and 200 µL of DMSO were applied to each well to solubilize the purple formazan crystals formed. The absorbance at 570 nm was measured using a plate spectrophotometer (Power Wave Xs, Bio-Tek, Winooski, VT, USA). Cell viability was presented as a percentage of untreated cells and non-irradiated cells (control cells) considered as 100%. Data represent the mean ± SD of four independent experiments. GraphPad Prism 7.0 (GraphPad Software, Inc., La Jolla, California, USA) was used for the statistical analysis. A comparison of values was performed with a non-parametric t test with Welch’s correction; p-values < 0.05 were used to define statistical significance.

### 2.15. Biodistribution Studies

Biodistribution of ^67^Ga-labelled AuNPs was evaluated in CD1 mice (obtained from Charles River, Barcelona, Spain). Animals were intravenously (i.v.) injected in the tail vein with the nanoparticles (1.5–6.0 MBq) diluted in 100 µL of NaCl 0.9%. Mice were sacrificed in groups of 3, by cervical dislocation at 1 h and 4 h after injection. The dose administered and the radioactivity in the sacrificed animals was measured using a dose calibrator (Capintec CRC25R). The difference between the radioactivity in the injected and sacrificed animals was assumed to be due to excretion. Tissues of interest were dissected, rinsed to remove excess blood, weighed, and their radioactivity was measured using a γ-counter (LB2111, Berthold, Germany). The uptake in the tissues was calculated and expressed as a percentage of the injected radioactivity dose per gram of tissue. The results are expressed as mean ± SD.

To assess the ability of the ^67^Ga-labelled AuNPs to be retained by tumors an additional set of experiments was carried out in 10–12 weeks old Balb/c-Nude mice with PC-3 xenografts, weighing approximately 16–20 g. A 150 µL bolus containing a suspension of approximately 7 × 10^6^ freshly harvested human PC-3 cells in Matrigel:PBS buffer 1:1 was subcutaneously injected in the right flank of each female nu/nu mouse. The animals were kept under aseptic conditions and 2 weeks later developed well-palpable tumors at the inoculation site. Xenograft-bearing animals were intratumorally injected with the ^67^Ga-labelled AuNPs in a small volume (20–30 µL of NaCl 0.9%) and were sacrificed at 1 h and 24 h after injection and tissue distribution was evaluated, as described for CD1 mice.

## 3. Results

### 3.1. Synthesis, Characterisation and Radiolabelling of the AuNPs

The AuNPs have been synthesized according to a previously described method [53]. Briefly, the 4–5 nm AuNP core, stabilized with TDOTA units, was complexed with GdCl_3_. The maximum Gd^3+^ coordination capacity was assessed by studying different Gd:AuNP ratios. After the complexation reactions, the resulting Gd-containing particles were purified by ultra-centrifugation. The AuNPs were washed with Milli-Q water until no free Gd^3+^ ion could be detected by colorimetric titration with xylenol orange [56]. Their Au and Gd^3+^ contents were determined by ICP-OES and it was found that the maximum content of gadolinium that could be incorporated into the nanoplatform corresponds to the ratio Au:Gd^3+^ 1:0.25 (Table 1). Thereafter, Gd-containing AuNPs (obtained using this ratio) were reacted with the thioctic acid bombesin derivative to obtain AuNP-Gd-BBN. The amount of BBN payload was determined by HPLC analysis, as detailed in the experimental section.

The particles AuNP-DOTA (without Gd^3+^ and without BBN), AuNP-Gd (with Gd^3+^ and without BBN) (using the AuNP:Gd 1:0.25 ratio) and AuNP-Gd-BBN (with Gd^3+^ and decorated with BBN) have been characterized by dynamic light scattering (DLS, Figure S2) and their zeta potential and molar ratios Au/Gd and Au/BBN determined (Table 2). Transmission electron microscopic (TEM) images were obtained for AuNP-DOTA and AuNP-BBN (Figure S3).

The final step to achieve multimodal imaging probes based on the AuNP-Gd and AuNP-Gd-BBN nanoplatforms involved their labeling with ^67^Ga, which was done by a reaction with ^67^GaCl_3_ at 70 °C within 30 min (>95% radiochemical yield).

The radiochemical stability of the ^67^Ga-radiolabeled AuNP-Gd was studied in 0.9% NaCl and in the presence of apo-transferrin, at 37 °C. The amount of radiolabeled nanoparticles was measured by ITLC radiochromatography at different time points (Figure S4).

### 3.2. Relaxometric Studies

**Figure 1:**
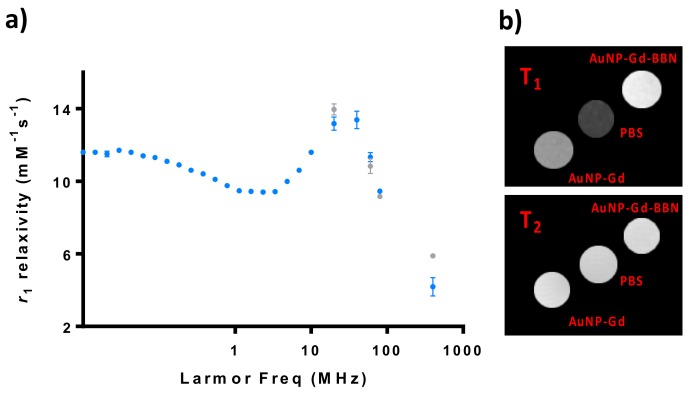
Relaxometric and MRI studies of AuNP-Gd and AuNP-Gd-BBN particles: (**a**) ^1^H NMRD profiles of AuNP-Gd (•) and AuNP-Gd-BBN (•) (25 °C); (**b**) T_1_- and T_2_-weighted MRI phantom images obtained at 9.4T (25 °C): [Gd]_AuNP-Gd_ = 0.25 mM and [Gd]_AuNP-Gd-BBN_ = 0.22 mM.

The ^1^H nuclear magnetic relaxation dispersion (NMRD) profile of particles with and without BBN has been studied and phantoms of their solutions were imaged at a 9.4 T MRI scanner (Figure 1). The particles containing BBN were only analyzed at higher fields (20–400 MHz).

The NMRD profiles of AuNP-Gd and AuNP-Gd-BBN present a hump at a magnetic field around 40 MHz, typical of nanoparticles, as can be verified in Figure 1. *r*_1_ and *r*_2_ relaxivities at 400 MHz were estimated from the image analysis of phantoms acquired at a 9.4 T MRI scanner. 

### 3.3. Cellular Internalization Studies

**Figure 2:**
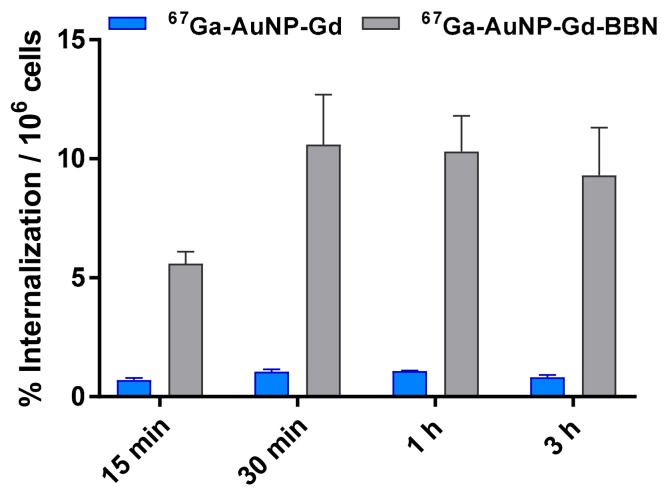
Cellular internalization studies of ^67^Ga-AuNP-Gd and ^67^Ga-AuNP-Gd-BBN in PC3 cells at 37 °C at different incubation times, determined by γ-counting. Results are expressed as the percentage of the total radioactivity internalized by the cells (mean ± SD; n = 3) and normalized per million of cells.

The cellular uptake of the ^67^Ga-labeled AuNPs was studied by the internalization rate in human prostate cancer PC3 cells (Figure 2) determined by a gamma-counting measurement of the radioactivity. The internalization of ^67^Ga-AuNP-Gd-BBN in PC3 cells was 6.5 ± 0.5, 10.6 ± 2.1, 10.3 ± 1.5, and 9.3 ± 2.0% of the total activity per million of cells at 15 min, 30 min, 1 h, and 3 h, respectively. Nonetheless, the internalization of ^67^Ga-AuNP-Gd in the same experimental conditions was 0.71 ± 0.08, 1.05 ± 0.08, 1.07 ± 0.02 and 0.81 ± 0.09% of the total activity per million of cells at the same time points.

### 3.4. Radiosensitization Studies

**Figure 3:**
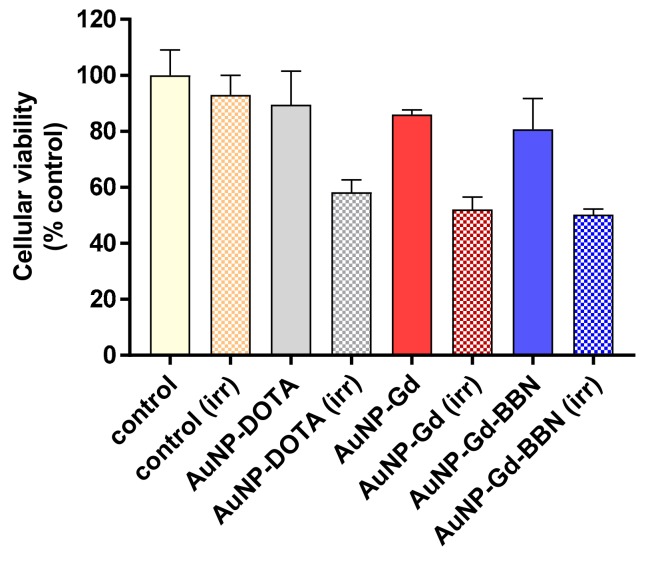
Cellular viability of PC3 cells treated with the different AuNPs (37.5 µg/mL) and γ-irradiated at 2 Gy (total dose). Cellular viability was determined by the MTT assay, 72 h after irradiation. Results are expressed as mean ± SD (n = 4).

The radiosensitizing capabilities of AuNP-DOTA, AuNP-Gd, and AuNP-Gd-BBN were evaluated in human prostate cancer PC3 cells, using the MTT colorimetric assay before and after γ irradiation of cells treated with the different nanoconstructs at different concentrations (0.6 to 75 µg/mL). Concerning the non-irradiated cells, no significant toxicity was observed after 72 h of incubation for all the concentrations tested (see Figure S5 and Table S1). As shown in Figure 3 (which displays results for cells incubated with 37.5 µg/mL), the exposure to γ radiation of the PC3 cells incubated with AuNP-DOTA induced a decrease in cellular viability of about 35% compared to control (irr) (58.2 ± 4.4 *vs* 93 ± 7%, *P* = 0.0002), whereas a slightly enhanced radiosensitizing effect was observed for AuNP-Gd (52.2 ± 4.0%) and AuNP-Gd-BBN (50.2 ± 2.0%), about 40% relative to the control (irr), but no significantly difference between them. While preliminary, these results indicate that the presence of Gd, another high Z element as Au, could contribute to the slight enhancement of the radiosensitizing effect (58.2 ± 4.4 vs. 52.2 ± 4.0%, *P* = 0.0444). Overall, the presence of both Gd and BBN upon irradiation induced a significant difference in cell viability: 58.2 ± 4.4 vs. 50.2 ± 2.0%, *P* = 0.0137.

### 3.5. Biodistribution Studies

**Figure 4:**
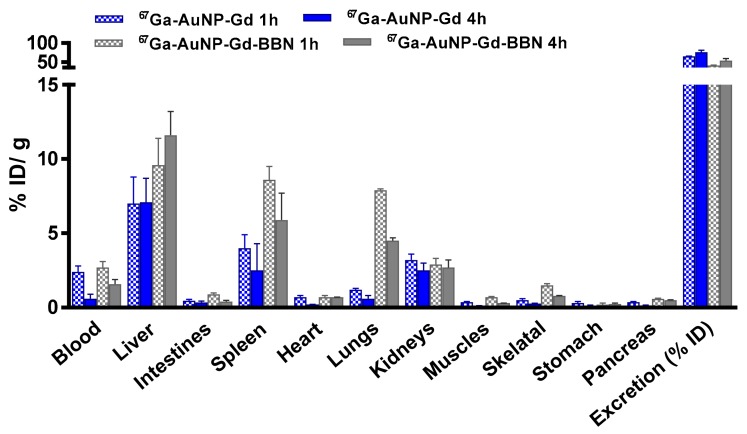
Biodistribution of ^67^Ga-AuNP-Gd and ^67^Ga-AuNP-Gd-BBN in CD1 mice, 1 h and 4 h after intravenous administration. Results are expressed as mean ± SD (n = 3).

**Figure 5:**
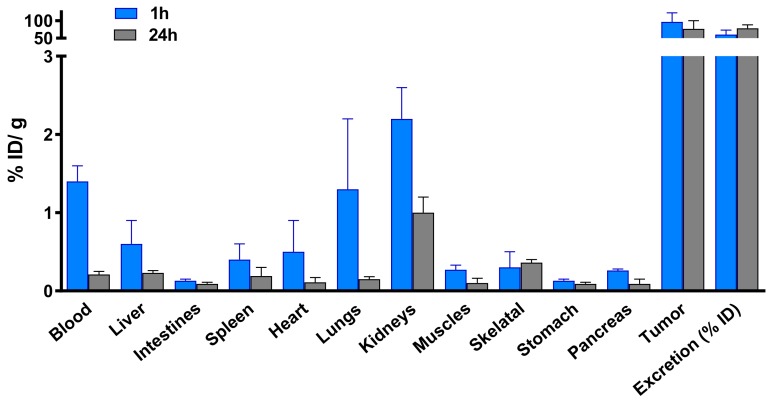
Biodistribution of ^67^Ga-AuNP-Gd-BBN in PC3 xenograft Balb/c mice, after 1 h and 24 h intratumoral administration. Results are expressed as mean ± SD (n = 3).

Biodistribution studies of ^67^Ga-AuNPs-Gd have been performed in CD1 mice, 1 h and 4 h after intravenous administration, to compare the tissue distribution profile (Figure 4, Table S2). To assess the ability of ^67^Ga-AuNP-Gd-BBN to be retained into the tumor mass, biodistribution studies were also performed in PC3 xenograft Balb/c mice, at 1 h and 24 h after intratumoral administration (Figure 5, Table S3). The main features of the tissue distribution profile of both ^67^GaAuNP-Gd preparations are the relatively fast blood clearance (2.4 ± 0.4 and 1.4 ± 0.5% ID/g at 1 and 4 h, respectively, for ^67^GaAuNP-Gd; 2.7 ± 1.2 and 1.6 ± 0.1% ID/g at 1 and 4 h, respectively, for ^67^GaAuNP-Gd-BBN) via both the hepatic and the renal pathways. Hepatic and splenic uptake was also found for both ^67^Ga labelled AuNPs. The overall rate of radioactivity excretion was moderate (< 65% for ^67^GaAuNP-Gd and < 55% for ^67^GaAuNP-Gd-BBN).

Biodistribution assessment of ^67^Ga-AuNP-Gd-BBN in PC3 xenograft Balb/c mice, at 1 h and 24 h after intratumoral administration, indicated a very low uptake in the main organs and a high tumor retention (96.5 ± 26.0 and 76.8 ± 23.3% ID/g at 1 and 24 h, respectively) (Figure 5).

**Figure 6:**
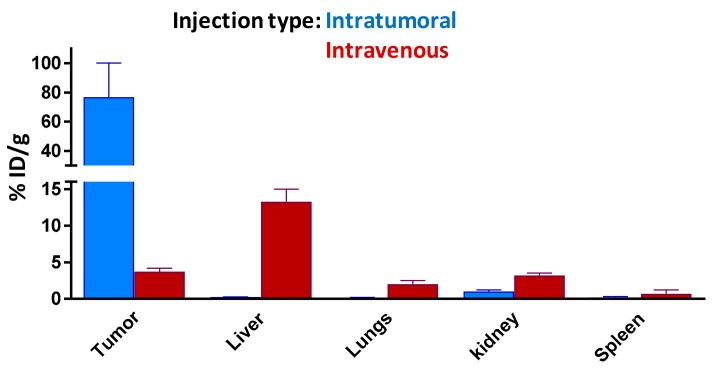
Comparison of tissue distribution of ^67^Ga-AuNP-Gd-BBN in PC-3 xenograft Balb/c mice, 24 h after intratumoral and intravenous administration. All data are expressed as % ID/g (mean ± SD, n = 3).

To confirm the targeting ability of our ^67^Ga-AuNP-Gd-BBN, the tumor uptake and tumor retention was also assessed in the same animal model after intravenous administration. In Figure 6, we compare the tumor uptake versus liver, spleen, kidney, and lung uptake 24 h after intratumoral and intravenous administration for a better understanding of the biodistribution profile of our AuNPs.

Data from this study clearly indicated that the AuNPs keep the ability to reach the tumor target. However, the radioactivity ratios of tumor to organs of the reticuloendothelial system (RES) highly depend on the route of administration.

## 4. Discussion

Gd-containing AuNPs were prepared by complexation of Gd^3+^ with AuNP-DOTA using different AuNP/Gd^3+^ ratios. This study revealed that the 1:0.25 ratio corresponds to the saturation of Gd^3+^ complexation (Table 1). Thus, the AuNP:Gd^3+^ obtained with the 1:0.25 ratio were further functionalized with a BBN thioctic acid derivative. Both types of AuNPs, carrying or not carrying the BBN derivative, have been fully characterized by different physico-chemical techniques. The results showed that the coordination of Gd^3+^ does not have a large influence on the hydrodynamic size nor on the zeta potential, but the presence of BBN does (Table 2, Figure S2). Indeed, the AuNP-Gd-BBN particles are bigger and have a less negative zeta potential. TEM images revealed a similar core size for AuNP-DOTA and AuNP-BBN (Figure S3), similar to our previously reported AuNPs [53].

The particles were then characterized regarding their potential application as MRI CAs. Their NMRD profile presents a hump at magnetic fields around 40 MHz, typical of nanoparticles with longer rotational correlation times (τ_R_) due to their slow mobility [12]. The presence of BBN has only a small influence on the relaxivity regardless of the 1.7-fold higher hydrodynamic diameter of the AuNP-Gd-BBN compared with the AuNP-Gd congeners.

At 20 MHz, we have measured *r*_1_ values of 13.17 and 13.95 mM^−1^·s^−1^ for AuNP-Gd and AuNP-Gd-BBN, respectively (25 °C). At 400 MHz (9.4 T), *r*_1_ values of 4.2 and 5.9 mM^−1^·s^−1^ and *r*_2_ values of 27.4 and 29.7 mM^−1^·s^−1^, were obtained for AuNP-Gd and AuNP-Gd-BBN, respectively. Comparison with the *r*_1_ relaxivities of the typical MRI GBCAs in clinical use (3.4 to 4.6 mM^−1^·s^−1^ at 1 T (42 MHz), 37 °C) [14,17,19,57,58], highlights the potential interest of these particles as T_1_-weighted MRI CAs.

Other gold NP-Gd reported in the literature have comparable *r*_1_ values: 14.6 mM^−1^·s^−1^ for Lip-DO3A@AuNP at 1.4 T (60 MHz) and 4.7 mM^−1^·s^−1^ at 7 T (300 MHz) [36], 12.9–15.6 mM^−1^·s^−1^ for the DO3A-based AuNP constructs at 1.4 T [57], 29–38 mM^−1^·s^−1^ at 0.47 T (20 MHz) and 10–12 mM^−1^·s^−1^ at 4.7 T (200 MHz) for the DO3A-aminopropionate-based AuNPs [59], and 14.6 mM^−1^·s^−1^, and an *r*_2_ of 38.5 mM^−1^·s^−1^ DNA-Gd@AuNP has an *r*_1_ of (measured at 1.4 T and 37 °C) [60].

The ratio *r*_2_/*r*_1_ obtained at 9.4 T is 6.6 for AuNP-Gd and 5.0 for AuNP-Gd-BBN. Typically, probes presenting a transverse-to-longitudinal relaxivity ratio (*r*_2_/*r*_1_) smaller than two are considered to be positive or T_1_-CAs. Ratios larger than 10 imply negative or T_2_- agents, while 2 < *r*_2_/*r*_1_ < 10 refers to potential dual-functional CAs [61,62]. Our nanoparticles can then be potentially used as dual MRI T_1_ and/or T_2_ agents. Figure 1b illustrates MRI T_1_- and T_2_-weighted images obtained from phantom tubes containing AuNPs prepared with similar Gd^3+^ content: [Gd]_AuNP-Gd_ = 0.25 mM and [Gd]_AuNP-Gd-BBN_ = 0.22 mM.

In vitro studies performed revealed that none of the AuNPs tested, in the range 1 to 160 µg/mL, exert significant cytotoxic activity on the PC3 human prostate tumor cancer cells, up to 24 h. The AuNPs functionalized with BBN appear slightly more cytotoxic than unmodified AuNPs, however without significant differences (Figure S1).

The internalization of particles containing both ^67^Ga and Gd was studied by gamma-counting measurement of ^67^Ga (over time, 15 min to 3 h) and by ICP measurement of Gd (only after 3 h of incubation). ^67^Ga-AuNP-Gd-BBN has shown a much higher level of internalization than ^67^Ga-AuNP-Gd (from 7.9 times to 11.5 times more at 15 min and 3 h, respectively), pointing out that the presence of BBN, as a targeting moiety towards the GRPr, has a strong influence on the cellular uptake of the nanoparticles. The results also show that the presence of Gd did not affect the uptake compared to the AuNPs previously studied [53]. Moreover, the Gd content in the cells incubated during 3 h with AuNP-Gd and AuNP-Gd-BBN, determined by ICP-OES, is in accordance; the same trend was observed, i.e., a higher level of Gd internalization for the AuNPs decorated with the BBN derivative. The Gd amount measured in the cell lysate after incubation with AuNP-Gd-BBN is approximately nine times higher than after incubation with AuNP-Gd. This allows us to conclude that the particles are internalized as a whole, confirming the in vitro stability of this nanoplatform.

Radiosensitization studies showed that the presence of the AuNPs induce radiotoxic effects, upon exposure of PC3 cells to a 2 Gy dose (γ-photons, 1530 keV), confirming that they have radiosensitizing properties. Reflecting the presence of gadolinium, a slightly enhanced radiosensitizing effect was observed for AuNP-Gd and AuNP-Gd-BBN, relative to the AuNP-DOTA and to the control. These results are in line with the well-recognized ability of Au and Gd to induce radiosensitizing effects by intensifying the generation of low-energy electrons (e.g., photoelectrons or Auger electrons) upon external irradiation of cells or tissues with photon or proton beams [5,26,32]. Due to the higher cellular internalization observed for the AuNP-Gd-BBN relatively to AuNP-Gd, promoted by the presence of the BBN peptide analogue, it was expected that those nanoparticles would induce additional cellular mortality [28,32,33]. However, the enhancement observed relatively to the congener AuNP-Gd was negligible, i.e., the efficacy of the cumulative effect of Au + Gd in some way is lost. Among other factors, the increased hydrodynamic size of the BBN-containing NPs (1.7-fold higher; Table 2) could therefore justify the loss of the expected cumulative effect Au + Gd. The differences in charge and coating of the NPs might also justify the effective dose enhancement induced by AuNP-Gd and AuNP-Gd-BBN. Our results are in agreement with other studies reported in the literature [5,26,28,32,33,63,64]. Overall, both AuNP-Gd and AuNP-Gd-BBN nanoplatforms have very promising properties as radiosensitizers.

Ex vivo biodistribution studies of ^67^Ga/Gd containing AuNPs were performed in CD1 control mice (1 and 4 h post injection) after intravenous administration to assess the potential of these multimodal AuNPs. The distribution found is in agreement with the pattern found for the AuNPs without Gd, previously reported, with little differences between preparations [53]. Uptake predominantly occurred in the liver and spleen, organs of the RES, a well-recognized parameter of the integrity of nanoparticles, also reported for other AuNPs, with different surface functionalization and similar core size, in control mice [36,57,65,66]. Thus, the results clearly attest to the high stability of these nanoplatforms in vivo. A small increase of the lung uptake was found in the biodistribution study of the ^67^Ga-AuNP-Gd-BBN due to the slightly higher hydrodynamic size of these nanoparticles. The kidney uptake (> 2.5% ID/g) and the high rate of excretion (> 50% ID at 4 h after administration) are in consonance with the small size of our AuNPs.

The ability of the ^67^Ga-AuNP-Gd-BBN to be retained in GRPr expressing tumors was evaluated in PC3-xenograft Balb/c mice at 1 and 24 h post injection (Figure 5). The high retention rate at 24 h confirmed the targeting capability of these radiolabeled AuNPs.

Biodistribution data indicated a low uptake in major organs associated to variable tumor, hepatic and splenic uptakes depending on the route of administration used. After 24 h post-administration, there is still a very high tumor uptake after intratumoral injection, while in the mice injected intravenously, an important fraction of the nanoparticles is retained in the organs of the reticuloendotelial system, in line with results described by others for different gold nanoparticles [65,66,67,68]. Overall, our studies suggest the potential usefulness of ^67^Ga-AuNP-Gd-BBN for molecular targeting of tumors expressing GRPr.

## 5. Conclusions

Herein, we present the successful synthesis and full characterization of novel bimodal (T_1_- and T_2_-) MRI/SPECT gold nanoparticles, with a high theranostic potential. In vitro studies evidence a good internalization in PC3 tumor cells of the particles when functionalized with a bombesin derivative. The radiosensitization studies revealed the dose enhancement potential of the combination of Au, Gd, and the targeting capability of such nanoconstructs.

Moreover, biodistribution evaluation of ^67^Ga-AuNP-Gd-BBN indicated a high lung, spleen, and liver uptake after intravenous administration in CD1 mice, which is in agreement with its high hydrodynamic size, while the intratumoral administration showed a high retention of the radioactivity in the tumour after 24 h post-injection.

Taking advantage of the versatility of DOTA coordination properties, our novel nanomaterial has a high theranostic potential; apart from the radiosensitization profile described herein, the ^67^Ga isotope can easily be replaced by other radiometals with therapeutic potential allowing for tuning of the AuNPs for the desired use. Indeed, the ^67^Ga could be replaced by ^68^Ga, ^90^Y, ^177^Lu, or ^165^Er, thus combining therapeutic with SPECT or PET as well as MR imaging potential of GRPr-positive tumors.

## Figures and Tables

**Table 1:** Gd and Au content of the NP corresponding to different complexation ratios, measured by ICP-OES.

Ratio AuNP:Gd	[Au] (mM)	[Gd] (mM)
1:0.05	0.452	0.025
1:0.10	0.442	0.042
1:0.25	0.467	0.245
1:0.50	0.068	0.229
1:5.00	0.045	0.183

**Table 2:** Hydrodynamic size and zeta potential (pH 6), Gd/Au, and BBN/Au ratios of the AuNPs.

Compound	Hydrodynamic Size (PDI) (nm)	Zeta Potential (mV)	Ratio (M) Au/Gd	Ratio (M) Au/BBN
AuNP-DOTA	30.9 (0.6)	−43.7 ± 13.1	---	---
AuNP-Gd^a^	47.3 (0.4)	−35.1 ± 14.1	1.9	---
AuNP-Gd-BBN^a^	78.8 (0.9)	−10.9 ± 5.1	3.2	4.1

^a^ AuNPs obtained using the AuNP:Gd 1:0.25 ratio.

## Supplementary Materials

The following are available online at https://www.mdpi.com/1996-1944/13/3/513/s1, Figure S1–S5 & Tables S1–S3: Figure S1. Effect of increasing concentrations of AuNPs on PC3 cells viability; Figure S2. DLS Size distribution diagrams of (a) AuNP-DOTA, (b) AuNP-Gd and (c) AuNP-Gd-BBN; Figure S3. TEM images with respective size histograms of (a) AuNP-DOTA and (b) AuNP-BBN; Figure S4. In vitro stability studies of ^67^Ga-AuNP-Gd under physiologic conditions and in the presence of apo-transferrin, performed by ITLC-SG chromatography and Figure S5. Cellular viability of PC3 cells treated with the different AuNPs at different concentrations and γ irradiated at 2 Gy (total dose). Table S1. Cellular viability of PC3 cells treated with the different AuNPs (37.5 µg/mL) and γ-irradiated at 2 Gy (total dose). Results are expressed as mean ± SD (n = 4); Table S2. Biodistribution results (mean ± SD, n = 3; expressed as % ID/g of organ) for ^67^Ga-AuNP-Gd and ^67^Ga-AuNP-Gd-BBN in CD1 mice, 1 h and 4 h after intravenous administration. Table S3. Biodistribution results (mean ± SD, n = 3; expressed as % ID/g of organ) for ^67^Ga-AuNP-Gd-BBN in PC3 xenograft Balb/c mice, after 1 h and 24 h intratumoral administration.
