# Supplementary material for: Dual Imaging Gold Nanoplatforms for Targeted Radiotheranostics
Source: Materials (Basel). 2020 Jan 22;13(3):513. doi: 10.3390/ma13030513 (PMC7040626; doi:10.3390/ma13030513)
Supplement: Supplementary file 1 [file materials-13-00513-s001.pdf]

materials

Dual Imaging Gold Nanoplatforms for Targeted Radiotheranostics

Francisco Silva [1,2], António Paulo [1,2], Agnès Pallier [3], Sandra Même [3], Éva Tóth [3], Lurdes Gano [1,2], Fernanda Marques [1,2], Carlos F.G.C. Geraldes [4,5,6], M. Margarida C.A. Castro [4,5], Ana M. Cardoso [7,8], Amália S. Jurado [4,7], Pilar López-Larrubia [9], Sara Lacerda [3,*] and Maria Paula Cabral Campello [1,2*]

1 Centro de Ciências e Tecnologias Nucleares, Instituto Superior Técnico, Universidade de Lisboa, Campus Tecnológico e Nuclear, Estrada Nacional 10, Km 139.7, 2695-066 Bobadela LRS, Portugal; fsilva@ctn.tecnico.ulisboa (F.S.); apaulo@ctn.tecnico.ulisboa.pt (A.P.); lgano@ctn.tecnico.ulisboa.pt (L.G.); fmarujo@ctn.tecnico.ulisboa.pt (F.M.)

2 Departamento de Engenharia e Ciências Nucleares (DECN), Instituto Superior Técnico, Universidade de Lisboa, Estrada Nacional 10, 2695-066 Bobadela LRS, Portugal

3 Centre de Biophysique Moléculaire, CNRS, UPR 4301, Université d'Orléans, Rue Charles Sadron, 45071 Orléans Cedex 2, France ; agnes.pallier@cnrs.fr (A.P.); sandra.meme@cnrs.fr (S.M.); eva.jakabtoth@cnrs-orleans.fr (E.T.)

4 Department of Life Sciences, Faculty of Science and TechnologyUniversity of Coimbra, Calçada Martim de Freitas, 3000-393 Coimbra, Portugal; geraldes@ci.uc.pt (C.F.G.C.G.); gcastro@ci.uc.pt (M.M.C.A.C.); asjurado@bioq.uc.pt (A.S.J.)

5 Coimbra Chemistry Center, University of Coimbra, Coimbra, Portugal

6 CIBIT/ICNAS Instituto de Ciências Nucleares Aplicadas à Saúde. Pólo das Ciências da Saúde, Azinhaga de Santa Comba, 3000-548 Coimbra, Portugal

7 CNC-Center for Neuroscience and Cell Biology, University of Coimbra, Coimbra, Portugal; amscardoso@ci.uc.pt

8 Institute for Interdisciplinary Research of the University of Coimbra, 3030-789 Coimbra, Portugal

9 Instituto de Investigaciones Biomédicas "Alberto Sols" CSIC/UAM, c/ Arturo Duperier 4, Madrid, Spain; plopez@iib.uam.es

* Correspondence: pcampelo@ctn.tecnico.ulisboa.pt (M.P.C.C.); sara.lacerda@cnrs.fr (S.L.)

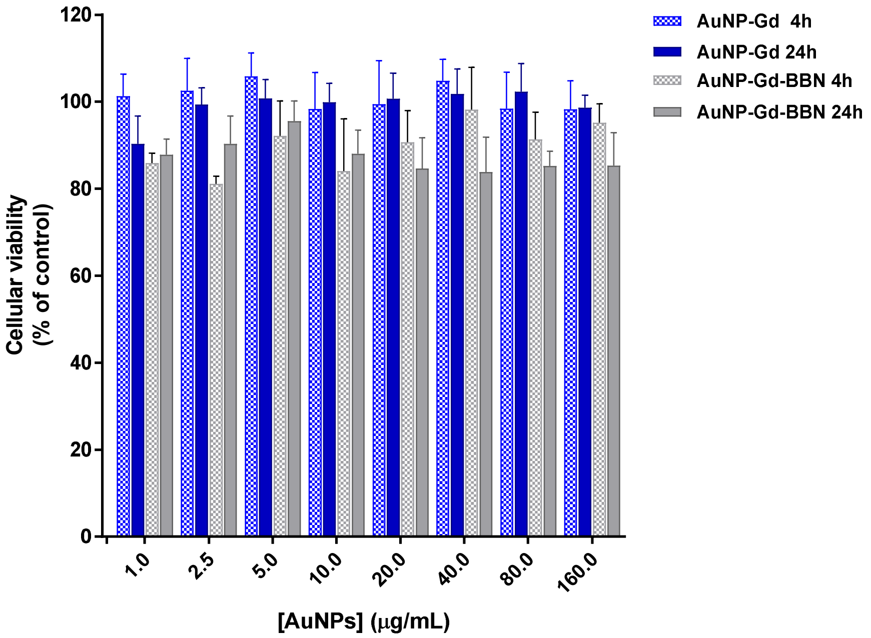

Figure 1. Effect of increasing concentrations of AuNPs on PC3 cells viability. PC3 cells were plated in 96-well plates at a density of 3 × 10^5 cells *per* well. Twenty-four hours after plating, the cells were incubated with 1, 2.5, 5, 10, 20, 40, 80 and 160 μg/mL solutions of AuNPs. Cell viability was measured after 4 or 24 h using the modified Alamar blue assay. Cell viability is presented relative to the percentage of untreated cells (control cells) considered as 100 %. Data represent the mean ± SD of four independent experiments.

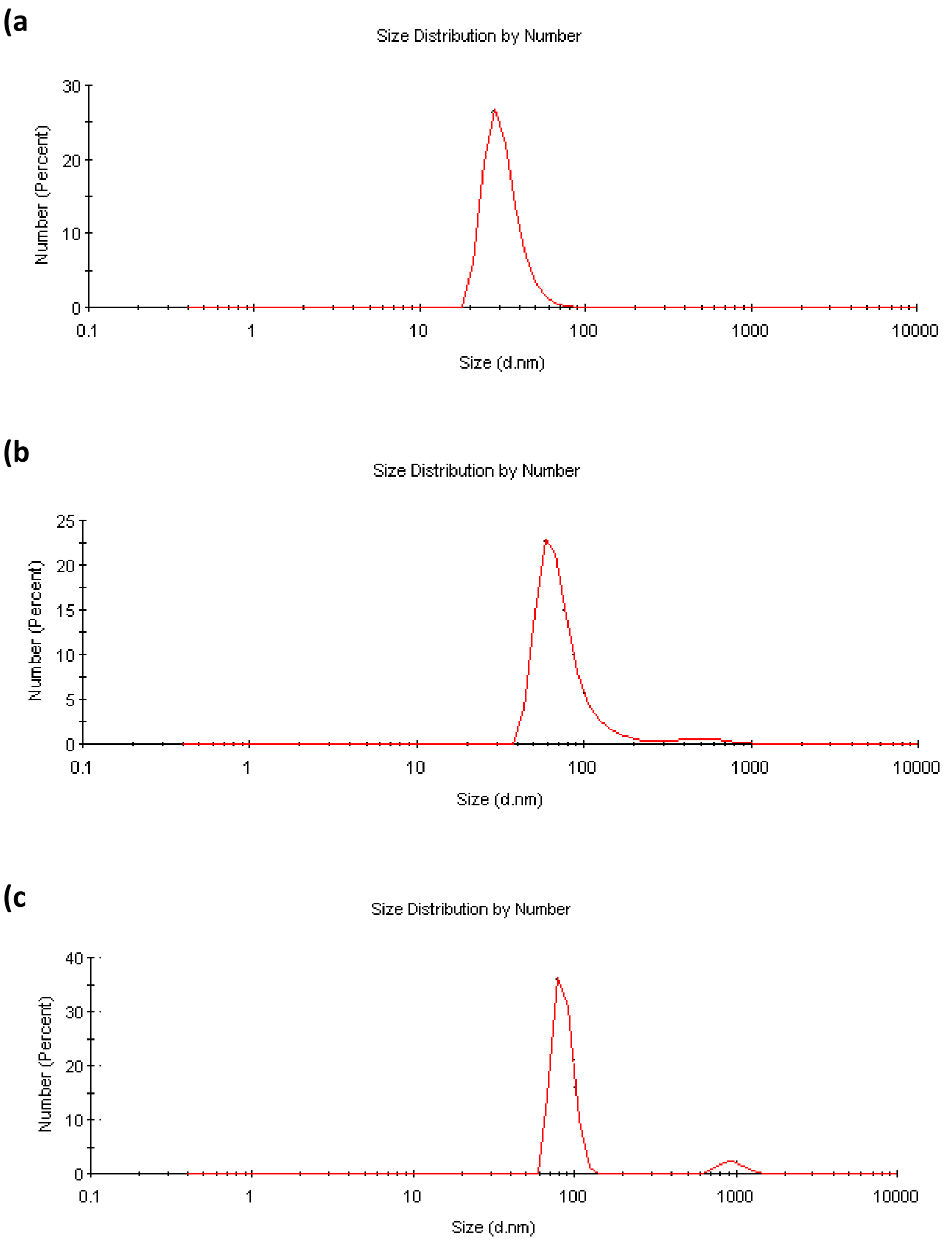

Figure 2. DLS Size distribution diagrams of (**a**) AuNP-DOTA, (**b**) AuNP-Gd and (**c**) AuNP-Gd-BBN.

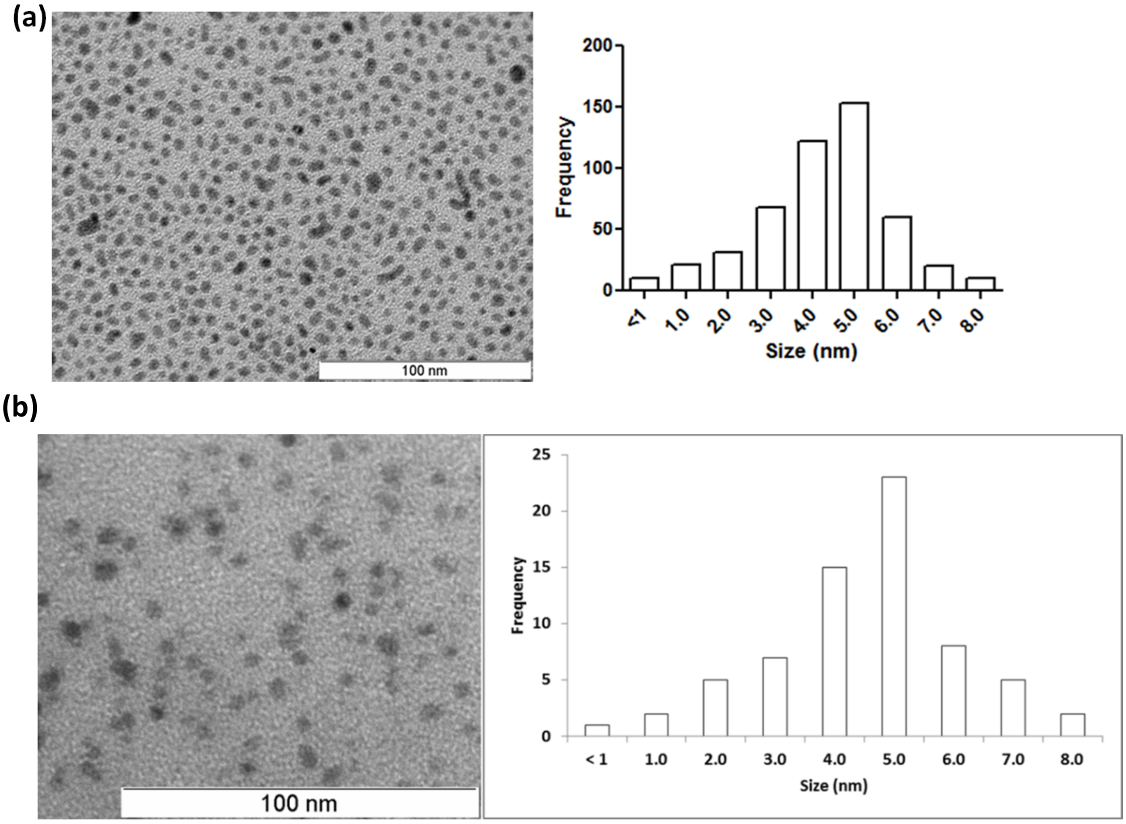

Figure 3. TEM images with respective size histograms of (**a**) AuNP-DOTA and (**b**) AuNP-BBN.

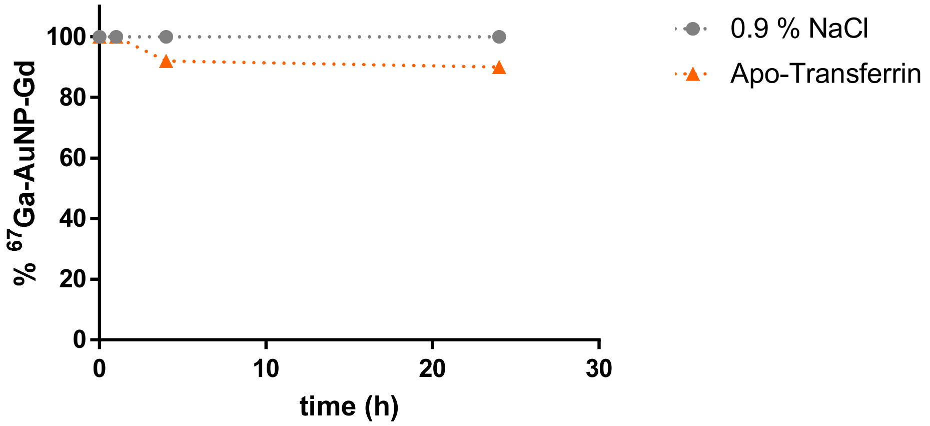

Figure 4. *In vitro* stability studies of ^{67}Ga-AuNP-Gd under physiologic conditions and in the presence of apo-transferrin, performed by ITLC-SG chromatography.

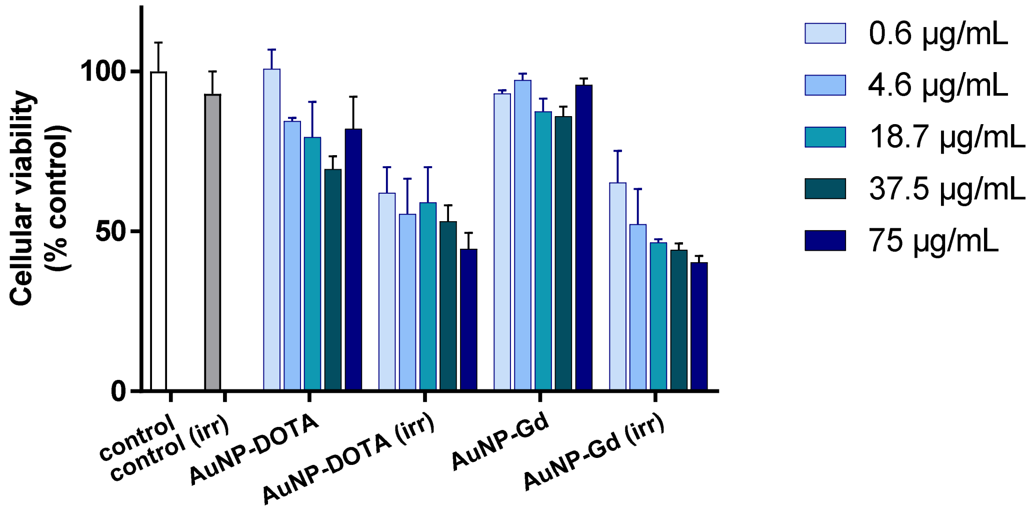

Figure 5. Cellular viability of PC3 cells treated with the different AuNPs at different concentrations (0.6, 4.6, 18.7, 37.5 and 75 µg/mL) and γ-irradiated at 2 Gy (total dose). Cellular viability was determined by the MTT assay, 72 h after irradiation. Data represent the mean ± SD (n = 4).

Table 1. Cellular viability of PC3 cells treated with the different AuNPs (37.5 µg/mL) and γ-irradiated (irr) at 2 Gy (total dose). Results are expressed as mean ± SD (n = 4).

Sample	cellular viability
Control	100.0 ± 9.0
Control (irr)	93.0 ± 7.0
AuNP	89.6 ± 12
AuNP (irr)	58.2 ± 4.4
AuNP-Gd	86.1 ± 1.6
AuNP-Gd (irr)	52.2 ± 4.0
AuNP-Gd-BBN	80.8 ± 11
AuNP-Gd-BBN (irr)	50.2 ± 2.0

Table 2. Biodistribution results (mean ± SD, n = 3; expressed as %ID/g of organ) for ^{67}Ga-AuNP-Gd and ^{67}Ga-AuNP-Gd-BBN in CD1 mice, 1 h and 4 h after intravenous administration.

Organ	^{67}Ga-AuNP-Gd		^{67}Ga-AuNP-Gd-BBN	
	1 h	4 h	1 h	4 h
Blood	2.40 ± 0.40	0.60 ± 0.30	2.70 ± 0.40	1.58 ± 0.30
Liver	7.00 ± 1.80	7.10 ± 1.60	9.60 ± 1.80	11.60 ± 1.60
Intestines	0.47 ± 0.09	0.35 ± 0.08	0.90 ± 0.09	0.40 ± 0.08
Spleen	4.00 ± 0.90	2.50 ± 1.80	8.60 ± 0.90	5.90 ± 1.80
Heart	0.70 ± 0.10	0.21 ± 0.01	0.70 ± 0.10	0.70 ± 0.01
Lungs	1.20 ± 0.10	0.60 ± 0.20	7.90 ± 0.10	4.50 ± 0.20
Kidneys	3.20 ± 0.40	2.50 ± 0.50	2.90 ± 0.40	2.70 ± 0.50
Muscles	0.36 ± 0.05	0.11 ± 0.01	0.70 ± 0.05	0.30 ± 0.01
Skeletal	0.50 ± 0.10	0.27 ± 0.02	1.50 ± 0.10	0.79 ± 0.02
Stomach	0.30 ± 0.10	0.12 ± 0.05	0.20 ± 0.10	0.25 ± 0.05
Pancreas	0.36 ± 0.05	0.14 ± 0.03	0.58 ± 0.05	0.49 ± 0.03
Excretion (%)	64.90 ± 0.50	75.90 ± 5.50	41.30 ± 0.50	53.60 ± 5.50

Table 3. Biodistribution results (mean ± SD, n = 3; expressed as %ID/g of organ) for ^{67}Ga-AuNP-Gd-BBN in PC3 xenograft Balb/c mice, after 1 h and 24 h intratumoral administration.

**Organ**	**^{67}Ga-AuNP-Gd-BBN**	
	**1 h**	**24 h**
Blood	1.40 ± 0.20	0.21 ± 0.04
Liver	0.60 ± 0.30	0.23 ± 0.03
Intestines	0.13 ± 0.02	0.09 ± 0.02
Spleen	0.40 ± 0.20	0.19 ± 0.11
Heart	0.50 ± 0.40	0.11 ± 0.06
Lungs	1.30 ± 0.90	0.15 ± 0.03
Kidneys	2.20 ± 0.40	1.00 ± 0.20
Muscles	0.27 ± 0.06	0.10 ± 0.06
Skeletal	0.30 ± 0.20	0.36 ± 0.04
Stomach	0.13 ± 0.02	0.09 ± 0.02
Pancreas	0.26 ± 0.02	0.09 ± 0.06
Tumor	96.5 ± 26.00	76.8 ± 23.30
Excretion (%)	60.00 ± 13.00	78.10 ± 9.80

© 2020 by the authors. Licensee MDPI, Basel, Switzerland. This article is an open access article distributed under the terms and conditions of the Creative Commons Attribution (CC BY) license (http://creativecommons.org/licenses/by/4.0/).
